# The Developing Social Brain: Implications for Education

**DOI:** 10.1016/j.neuron.2010.03.004

**Published:** 2010-03-25

**Authors:** Sarah-Jayne Blakemore

**Affiliations:** 1 Institute of Cognitive Neuroscience, University College London, 17 Queen Square, London WC1N 3AR, UK

## Abstract

This paper discusses the development of the human social brain. First, I will argue that social cognition is uniquely important and describe evidence that social interaction plays a critical role in early brain development. I will then discuss recent research demonstrating that the social brain undergoes protracted development and that adolescence in particular represents a period of reorganization of the social brain. Finally, I will attempt to draw out potential implications of this new research for education policy and for human wellbeing.

## Main Text

### Humans Are Exquisitely Social

**Figure 1:**
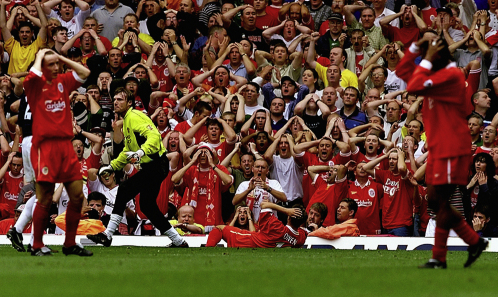
Owen Misses a Goal Phil Noble/Press Association.

Humans are an exquisitely social species. Take the photograph shown here (Figure 1), which shows an English soccer player, Michael Owen, having just missed a goal for Liverpool Football Club. The photograph beautifully illustrates two aspects of the social brain. First, it shows how rapid and instinctive social responses are. Within a split second of Michael Owen missing the goal, nearly everyone is making identical arm gestures and has the same expression on their face. The other aspect of the social brain this photograph illustrates is our ability to read other people's gestures and faces in terms of their underlying emotions and mental states. Without having to ask you have a good idea of what they are thinking and feeling at this precise moment in time.

We are constantly reading each others' actions, gestures and faces in terms of underlying mental states and emotions, in an attempt to figure out what other people are thinking and feeling, and what they are about to do next. This is known as theory of mind or mentalizing. Developmental psychology research on theory of mind has demonstrated that the ability to understand others' mental states develops over the first four or five years of life. While certain aspects of theory of mind are present in infancy (Baillargeon et al., 2010), it is not until around the age of four years that children begin explicitly to understand that someone else can hold a belief that differs from one's own and which can be false (Barresi and Moore, 1996). An understanding of others' mental states plays a critical role in social interaction because it enables us to work out what other people want and what they are about to do next and to modify our own behavior accordingly.

### Social Cognition Is Special

It sounds obvious to say that interaction with other people is critical for normal neurocognitive development. However, there is a striking and surprising empirical example of the importance of social interaction for learning from research on language acquisition. It is well known that many Japanese people are unable to distinguish between R and L sounds. However, research in the 1980s revealed that Japanese babies are able to detect the difference between R and L, but only before about nine months (e.g., Werker, 1989). The Japanese language does not contain distinct R and L sounds, so Japanese babies are not exposed to the subtle difference between these sounds and eventually lose the ability to distinguish them by the age of nine months.

**Figure 2:**
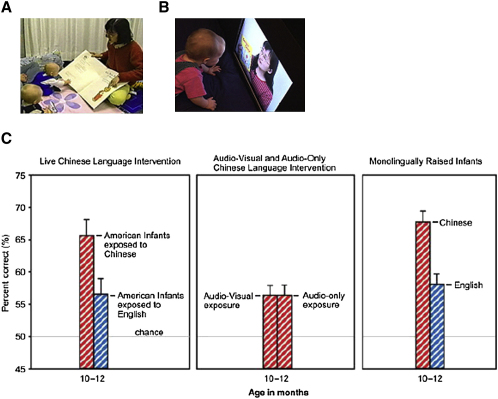
Learning Language Sounds Infants were exposed to Mandarin via live interaction with a native Mandarin speaker (A) or via audio-visual (B) or audio-only (not shown). A control group had live exposure to language but heard only English. After exposure, all infants were tested with two Mandarin Chinese sounds. Results indicate learning in the live exposure group, but not in the TV or audio-only groups (C) (from Kuhl et al., 2003).

A key question is whether sounds that have been lost can be relearned. A study confirmed that infants older than nine months can learn to discriminate speech sounds to which they have not previously been exposed (Kuhl et al., 2003). Kuhl and colleagues studied American babies who had grown up hearing only English and had thus lost the ability to distinguish between certain Chinese Mandarin sounds (Figure 2). The authors trained three groups of nine-month-old American babies: one group interacted with a real native Chinese speaker, who played with and read to them; a second group saw movies of the same Chinese speaker; the third group heard the same Chinese speaker through headphones. The content and the time of exposure were identical in all three groups.

The group that had been exposed to a real live Chinese person significantly improved their ability to distinguish between the two sounds, performing at around the same level as native Chinese babies. In striking contrast, babies who had been exposed to the same amount of Chinese but in the form of video or sound recordings showed no learning, and their posttraining performance was the same as the American babies who had received no exposure.

There are two implications of this research. First, it shows that relearning is possible. Although zero to nine months represents a sensitive period for sound categorization, it is possible to relearn sounds after this window of opportunity has closed. Second, the training results show that social interaction is a critical and constraining factor. There appears to be something special about social interaction with a real live person that is not present from watching videos or hearing sound recordings of the same person. What is special about social interaction with a real person is not yet understood. One possibility is that social interaction increases infants' motivation through enhanced attention and arousal. Social interaction also directs the adult trainer to focus on the learner's individual needs and tailors the training content for the learner. In addition, by nine months, infants start to understand that pointing to, or looking in the direction of, an object indicates that this object is being referred to. This is one of the first building blocks of theory of mind.

### The Social Brain

Over the past 15 years, a large number of independent studies have shown remarkable consistency in identifying the brain regions that are involved in theory of mind or mentalizing. These studies have employed a wide range of stimuli including stories, sentences, words, cartoons, and animations, each designed to elicit the attribution of mental states (see Amodio and Frith, 2006, for review). In each case, the mentalizing task resulted in the activation of a network of regions including the posterior STS at the temporoparietal junction (TPJ), the temporal poles and the dorsal medial PFC (mPFC; see Burnett and Blakemore, 2009). The agreement between neuroimaging studies in this area is remarkable, and the consistent localization of activity within a network of regions including the pSTS/TPJ and mPFC, as well as the temporal poles, suggests that these regions are key to the process of mentalizing.

Brain lesion studies have consistently demonstrated that the superior temporal lobes (e.g., Samson et al., 2004) and PFC (e.g., Stuss et al., 2001) are involved in mentalizing as damage to these brain areas impairs mentalizing abilities. Interestingly, one study reported a patient with large PFC damage whose mentalizing abilities were intact (Bird et al., 2004), suggesting that this region is not necessary for mentalizing. However, there are other explanations for this surprising and intriguing finding. It is possible that, due to plasticity, this patient used a different neural strategy in mentalizing tasks. Alternatively, it is possible that damage to this area at different ages has different consequences for mentalizing abilities. The patient described by Bird and colleagues had sustained her PFC lesion at a later age (62 years) than most previously reported patients who show impairments of mentalizing tasks. Perhaps mPFC lesions later in life spare mentalizing abilities, whereas damage that occurs earlier in life is detrimental. It is possible that mPFC is necessary for the acquisition of mentalizing but not essential for later implementation of mentalizing. Intriguingly, this is in line with recent data from developmental fMRI studies of mentalizing, which suggest that the mPFC contributes differentially to mentalizing at different ages.

#### Development of Mentalizing during Adolescence

**Figure 3:**
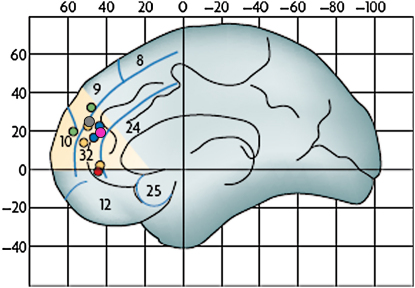
Medial Prefrontal Activation Decreases during Adolescence A section of the dorsal MPFC that is activated in studies of mentalizing is shown between red lines: Montreal Neurological Institute (MNI) y coordinates range from 30 to 60, and z coordinates range from 0 to 40. Colored dots indicate voxels of decreased activity during six mentalizing tasks between late childhood and adulthood (see Blakemore, 2008, for references). The mentalizing tasks ranged from understanding irony, which requires separating the literal from the intended meaning of a comment (green dots), thinking about one's own intentions (blue dots), thinking about whether character traits describe oneself or another familiar other (yellow dots; also Pfeifer et al., 2009; gray dot), watching animations in which characters appear to have intentions and emotions (red dot) and thinking about social emotions such as guilt and embarrassment (Burnett et al., 2009; pink dot). (Adapted from Blakemore, 2008).

A number of developmental fMRI studies of mental state attribution have consistently shown that mPFC cortex activity during mentalizing tasks decreases between adolescence and adulthood (Figure 3). Each of these studies compared brain activity in young adolescents and adults while they were performing a task that involved thinking about mental states (see Figure 3 for details of studies). In each of these studies, mPFC activity was greater in the adolescent group than in the adult group during the mentalizing task compared to the control task. In addition, there is evidence for differential functional connectivity between mPFC and other parts of the mentalizing network across age (Burnett and Blakemore, 2009).

To summarize, a number of developmental neuroimaging studies of social cognition have been carried out by different labs around the world, and there is striking consistency with respect to the direction of change in mPFC activity. It is not yet understood why mPFC activity decreases between adolescence and adulthood during mentalizing tasks, but two non-mutually exclusive explanations have been put forward (see Blakemore, 2008, for details). One possibility is that the cognitive strategy for mentalizing changes between adolescence and adulthood. A second possibility is that the functional change with age is due to neuroanatomical changes that occur during this period. Decreases in activity are frequently interpreted as being due to developmental reductions in gray matter volume, presumably related to synaptic pruning. However, there is currently no direct way to test the relationship between number of synapses, synaptic activity, and neural activity as measured by fMRI in humans (see Blakemore, 2008, for discussion). If the neural substrates for social cognition change during adolescence, what are the consequences for social cognitive behavior?

#### Online Mentalizing Usage Is Still Developing in Mid-adolescence

Most developmental studies of social cognition focus on early childhood, possibly because children perform adequately in even quite complex mentalizing tasks at around age four. This can be attributed to a lack of suitable paradigms: generally, in order to create a mentalizing task that does not elicit ceiling performance in children aged five and older, the linguistic and executive demands of the task must be increased. This renders any age-associated improvement in performance difficult to attribute solely to improved mentalizing ability. However, the protracted structural and functional development in adolescence and early adulthood of the brain regions involved in theory of mind might be expected to affect mental state understanding.

**Figure 4:**
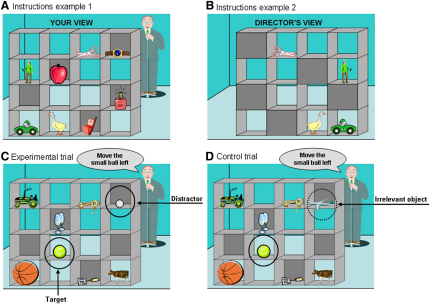
The Shelves Task (A and B) Images used to explain the Director condition: participants were shown an example of their view (A) and the corresponding director's view (B) for a typical stimulus with four objects in occluded slots that the director cannot see (e.g., the apple). (C and D) Example of an Experimental (C) and a Control trial (D) in the Director condition. The participant hears the verbal instruction: “Move the small ball left” from the director. In the Experimental trial (C), if the participant ignored the director's perspective, she would choose to move the distractor ball (golf ball), which is the smallest ball in the shelves but which cannot be seen by the director, instead of the larger ball (tennis ball) shared by both the participant's and the instructor's perspective (target). In the Control trial (D), an irrelevant object (plane) replaces the distractor item. (Adapted from Dumontheil et al., 2010).

Recently, we adapted a task that requires the online use of theory of mind information when making decisions in a communication game and which produces large numbers of errors even in adults (Keysar et al., 2003). In our computerized version of the task, participants view a set of shelves containing objects, which they are instructed to move by a “Director,” who can see some but not all of the objects (Dumontheil et al., 2010; Figure 4). Correct interpretation of critical instructions requires participants to use the director's perspective and only move objects that the director can see. We tested participants aged between 7 and 27 years and found that, while performance in the director and a control condition followed the same trajectory until mid-adolescence, the mid-adolescent group made more errors than the adults in the director condition only. These results suggest that the ability to take another person's perspective to direct appropriate behavior is still undergoing development at this relatively late stage.

### Implications for Education

Knowledge of how the brain develops and learns will have a profound impact on education in the future. Understanding the brain mechanisms that underlie learning and memory, and the effects of genetics, the environment, emotion, and age on learning could transform educational strategies and enable us to design programs that optimize learning for people of all ages and of all needs. Neuroscience can now offer some understanding of how the brain learns new information and processes this information throughout life (see Blakemore and Frith, 2005).

As described above, social interaction with a real live person is critical for at least some types of early learning (Kuhl et al., 2003), suggesting that, while not necessarily harmful, DVDs and CDs aimed at teaching babies and young children may not be associated with optimal learning. More importantly, the time spent watching DVDs is time that could otherwise be spent in social interaction with a real person, and denying the developing brain of this might have negative consequences. We need to ask whether online social networking, which is particularly popular with teenagers, is the same as real live interaction, or whether it might be denying the developing teenage brain of important real life interactions. There is as yet no research on this important question. What is the critical factor in social interaction that is so evidently missing from video conferencing, and which makes it incomparable to a meeting with real people? There is a growing industry for the development of robot nannies, robot carers, and robot companions for the elderly in aging societies such as Japan. But are robot companions the same as real friends? Does social interaction with robots determine happiness in the same way as social relationships with people (Argyle, 2001)? These are open questions, ripe for research.

Understanding the brain basis of social functioning and social development is crucial to the fostering of social competence inside and outside the classroom. Social functioning plays a role in shaping learning and academic performance (and vice versa), and understanding the neural basis of social behavior can contribute to understanding the origins and process of schooling success and failure. The finding that changes in brain structure continue into adolescence (and beyond) has challenged accepted views and has given rise to a recent spate of investigations into the way cognition (including social cognition) might change as a consequence. Research suggests that adolescence is a key time for the development of regions of the brain involved in social cognition and self-awareness. This is likely to be due to the interplay between a number of factors, including changes in the social environment and in puberty hormones, as well as structural and functional brain development and improvements in social cognition.

If early childhood is seen as a major opportunity—or a “sensitive period”—for teaching, so too should the teenage years. During both periods, particularly dramatic brain reorganization is taking place. The idea that teenagers should still go to school and be educated is relatively new. And yet the research on brain development suggests that education during the teenage years is vital. The brain is still developing during this period, is adaptable, and needs to be molded and shaped. Perhaps the aims of education for adolescents might change to include abilities that are controlled by the parts of the brain that undergo most change during adolescence. These abilities include internal control, multitasking, and planning—but also self-awareness and social cognitive skills such as the perspective-taking and the understanding of social emotions. Finally, it might be fruitful to include in the curriculum some teaching on the changes occurring in the brain during adolescence. Adolescents might be interested in, and could benefit from, learning about the changes that are going on in their own brains.
